# Assessment of intratumoral collagen in pituitary neuroendocrine tumors (pituitary adenomas/PitNets) via digital pathology and its correlation with intraoperative consistency

**DOI:** 10.1007/s11102-026-01703-8

**Published:** 2026-05-23

**Authors:** Acitores Cancela A., Ayuso-Cerezo H., Rodríguez Berrocal V., Carretero-Barrio I., Ruz-Caracuel I., Pian H., Díez Juan J., Iglesias P.

**Affiliations:** 1 Department of Neurosurgery, Hospital Universitario Ramón y Cajal, Madrid, Spain; 2 Department of Neurosurgery, Hospital Universitario HM Puerta del Sur, Madrid, Spain; 3 Department of Medicine, Universidad Autónoma de Madrid, Madrid, Spain; 4 Faculty of Sciences, Universidad de Alcalá, Alcalá de Henares, Madrid, Spain; 5 Pathology Department, Hospital Universitario Ramón y Cajal, IRYCIS, Madrid, Spain; 6 Centre for Biomedical Research in Cancer Networks (CIBERONC), Carlos III Health Institute, Madrid, Spain; 7 Faculty of Medicine, Universidad de Alcalá, Alcalá de Henares, Madrid, Spain; 8 Department of Endocrinology, Hospital Universitario Puerta de Hierro Majadahonda, Majadahonda, Spain; 9 Instituto de Investigación Sanitaria Puerta de Hierro Segovia de Arana, Majadahonda, Spain

**Keywords:** Pituitary neuroendocrine tumors (pituitary adenomas/PitNets), Tumor consistency, Collagen quantification, Digital pathology, Endoscopic transsphenoidal surgery

## Abstract

**Introduction:**

The success of endoscopic transsphenoidal resection of pituitary neuroendocrine tumors (pituitary adenomas/PitNets) depends largely on tumor consistency, as fibrous tumors pose greater surgical difficulty and are associated with higher morbidity. However, histological correlates of intraoperative consistency remain inconsistently defined. This study evaluated the relationship between intraoperative tumor consistency and collagen content using digital image analysis.

**Methods:**

Biopsy samples from patients undergoing surgery for pituitary adenomas/PitNets were included. Tumor consistency was assessed intraoperatively by the same neurosurgeon and classified as soft, firm, or fibrous. Histological sections were stained with Masson’s trichrome, and digital image analysis was performed using QuPath software. A trained pixel classifier artificial neural network differentiated collagen, tumor tissue, and background. Collagen content, as a surrogate marker of fibrosis, was quantified as a percentage of total tumor area. All analyses were conducted blinded to intraoperative assessments.

**Results:**

A total of 69 tumors were analyzed: 30 soft (43.5%), 22 firm (31.9%), and 17 fibrous (24.6%). Median collagen content increased progressively across groups (6.3%, 8.3%, and 22.0%, respectively). Fibrous tumors demonstrated significantly higher collagen content compared with soft and firm tumors (*p* < 0.05), while no significant difference was observed between soft and firm tumors. Collagen content was not associated with tumor subtype or patient age, and its deposition was heterogeneous between paired tissue blocks (CV = 59.83%).

**Conclusion:**

Digital collagen quantification provides an objective histological correlate of intraoperative tumor consistency, validating the distinction between soft and fibrous tumors, and establishing a reference standard for future studies aimed at developing preoperative predictive models.

## Introduction

Pituitary neuroendocrine tumors (pituitary adenomas/PitNets), also referred to as pituitary adenomas, are among the most common neoplasms of the central nervous system [1]. Surgery is the main treatment for most subtypes, except for prolactinomas, which generally respond to dopamine agonists [2–4]. The endoscopic endonasal transsphenoidal approach has largely replaced traditional microsurgical and transcranial techniques in specialized centers.

Tumor consistency has gained increasing attention because of its direct surgical implications. Most classifications distinguish soft tumors, which are readily aspirated, from fibrous lesions, which require fragmentation and specialized instrumentation [5–11]. Correctly identifying fibrous tumors preoperatively is essential, as they are associated with significantly lower complete resection rates (45.8% vs. 76.2%) and a markedly higher risk of incomplete removal (odds ratio 7.13), with consequent complications such as cerebrospinal fluid (CSF) leak, permanent diabetes insipidus, or panhypopituitarism [9, 12]. Identifying fibrous tumors preoperatively is essential, as it directly impacts surgical planning, patient counselling, and the decision to refer to a specialized center. However, predicted fibrous consistency should be understood as one factor within a broader risk stratification framework, not the sole criterion for centralization. International guidelines demonstrate that outcomes are superior across all pituitary adenoma subtypes when managed at high-volume Pituitary Tumor Centers of Excellence. Preoperative consistency prediction therefore serves to refine patient-center matching, adding weight to the case for referral when combined with other complexity factors, with the ultimate goal of ensuring that every patient is treated in the environment best equipped for their individual risk profile [13, 14].

Histopathological studies have linked fibrous tumor consistency to increased collagen deposition. Although hematoxylin-eosin and Masson trichrome stains provide useful information, the relationship between fibrosis and tumor consistency has been predominantly assessed subjectively in the existing literature. Moreover, traditional qualitative or manually quantitative approaches are limited by heterogeneous collagen distribution, processing artifacts, analysis of selected microscopic fields rather than the entire slide, and operator-dependent variability. To our knowledge, this is the first study to apply a digital pathology-based approach to objectively quantify collagen content across whole slides, providing a comprehensive and reproducible assessment of the histologic substrate underlying tumor consistency [6, 15–20].

Digital pathology combined with artificial intelligence offers a more objective alternative. This enables consistent and reproducible quantification of intratumoral collagen, overcoming the limitations of manual evaluation. However, the usefulness of these techniques for establishing a comprehensive and clinically relevant quantification of tumor fibrosis in pituitary adenomas/PitNets has not yet been defined, underscoring the need to systematically evaluate their relationship with intraoperative consistency.

## Materials and methods

### Patient selection

Patients who underwent surgery at Hospital Universitario Ramón y Cajal between 2008 and 2023 were included in the study. All procedures involved endoscopic transsphenoidal surgery performed by the same neurosurgeon from the Department of Neurosurgery (VRB). Cases with diagnoses other than pituitary adenomas/PitNets, insufficient tissue samples, or unretrievable specimens were excluded. The study received approval from the institutional ethics committee (approval date: October 4, 2019, Code: Acta 372).

### Consistency classification

Tumor consistency was assessed intraoperatively by the senior operating neurosurgeon, as previously described [10, 21]. Pituitary adenomas/PitNets were classified into three categories according to their surgical handling characteristics: “soft”, defined as tumors that could be easily aspirated using an 8–10 Fr suction tip; “firm”, referring to lesions requiring limited fragmentation or manipulation with ring curettes; and “fibrous”, corresponding to tumors necessitating extensive manipulation with ring curettes, the use of sharp instruments for debulking, and, in some cases, extracapsular dissection to achieve complete removal [9, 22].

### Histological processing and digital analysis

A neuropathologist reviewed all cases to confirm the pituitary adenomas/PitNets diagnosis. Histopathological subtyping was performed according to WHO 5th edition [23] by performing immunohistochemical studies of hormones and pituitary transcription factors: prolactin (EP193, Vitro), growth hormone (polyclonal, Leica), follicle stimulating hormone (INN-hFSH-60, Leica), luteinizing hormone (BSB-53, Bio SB), thyroid stimulating hormone (EP254, Vitro), adrenocorticotrophic hormone (polyclonal, Leica), PIT-1 (D7, Santa Cruz), SF1 (EPR19744, Abcam), TPIT (polyclonal, Vitro); and keratin CAM 5.2 (CAM5.2, Cell Marque).

Pituitary adenomas/PitNets were stained using a commercial Masson’s trichrome kit (Ventana Medical Systems, Roche Diagnostics) on a BenchMark Special Stains apparatus (Ventana Medical Systems, Roche Diagnostics). This stain differentiates between collagen (blue/green), nuclei (black/gray) and cytoplasm (pink/red). Histopathological sections were digitalized using a Philips UltraFast Scanner^®^, and the resulting whole slide images (WSI) were downloaded.

Digital analysis was performed using QuPath software [24]. A representative area of the sample including tumor, collagen, and background is selected to estimate the stain vectors by an integrated algorithm that deconvolutes the colors. This enables the software to specifically identify the contribution of each dye within the sample, thereby reducing the possible color bias between different pituitary adenomas/PitNets analyses.

**Fig. 1:**
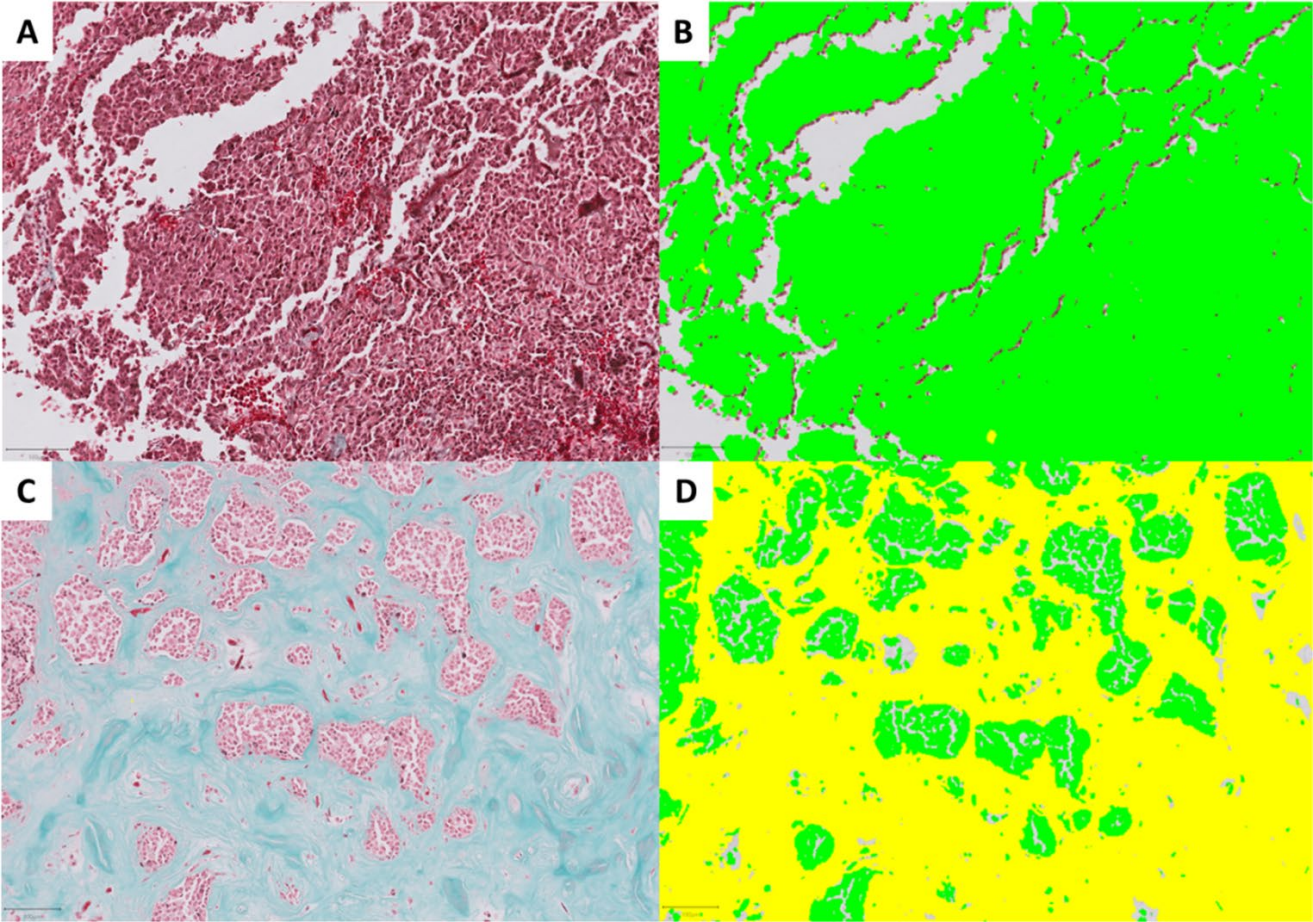
(**A**) Masson’s trichrome stained pituitary adenoma/PitNet with soft intraoperative consistency. (**B**) Real-time pixel-level classification of the same case. (**C**) Masson’s trichrome stained pituitary adenoma/PitNet with fibrous intraoperative consistency. (**D**) Real-time pixel-level classification of the same case. Pixels classified as tumor are represented as green, and collagen pixels as yellow. Scale bar: 100 μm

Tumor areas were manually annotated and validated by a certified neuropathologist in QuPath, while non-neoplastic tissue and artifacts were excluded. The built-in QuPath pixel classificator was then used to classify every pixel of the WSI into one of three categories: tumor, collagen or background. This pixel classifier is based on a multilayer perceptron artificial neural network (ANN_MLP). The training is built upon at least three tumor, collagen, and background examples, considering the sample size and possible color variations within it. Nevertheless, the classification results were reviewed in real time, providing new training examples when needed. Finally, the software provides the area occupied by each category inside the tumor area previously annotated. The analysis was conducted blindly, without prior knowledge of intraoperative consistency (Fig. 1).

### Establishment of a subcohort for inter-block variability assessment and methodological reproducibility

To assess concordance in collagen quantification across tissue blocks from the same case, a second FFPE block was selected from cases in which more than one FFPE block was available. A neuropathologist reviewed all new selected blocks to confirm the presence of pituitary adenoma/PitNET. The composition of this subcohort was designed to reflect the relative frequencies of intraoperative consistency categories (soft, firm, and fibrous) observed in the primary cohort, ensuring a representative sample for variability analysis.

To evaluate the reproducibility of the collagen quantification methodology, a randomly selected subset of pituitary adenoma/PitNET samples from the primary cohort underwent repeated analysis (retest).

In both the inter-block variability analysis and the retest, histological processing and digital image analysis were performed following the same protocol described in the Histological Processing and Digital Analysis section, and all analyses were carried out by the same operator. This approach minimizes measurement errors and eliminates inter-observer variability as a confounding factor.

### Statistical analysis

Normality of the distribution of quantitative variables was assessed using the Shapiro-Wilk test, while homoscedasticity was evaluated with Levene’s test. Quantitative variables are expressed as mean ± standard deviation (SD) or as median and interquartile range (IQR), as appropriate. Nonparametric comparisons of fibrosis percentages across consistency groups were conducted using the Kruskal-Wallis test, followed by Dunn’s post hoc test. Categorical variables were analyzed using either the chi-square test or Fisher’s exact test, and Pearson’s correlation coefficient (r) was used to assess the relationship between age and fibrosis.

The diagnostic accuracy of collagen quantification for predicting tumor consistency was evaluated using Receiver Operating Characteristic (ROC) curve analysis. Given the non-normal distribution of the data, a non-parametric approach was employed. The Area Under the Curve (AUC) was calculated to assess overall discriminative performance, and the optimal diagnostic threshold was identified using the Youden Index (J), maximizing the balance between sensitivity and specificity to differentiate firm from non-firm (soft and intermediate) pituitary adenomas/PitNETs.

Intratumoral variability in collagen quantification between paired FFPE tissue blocks was assessed using the Coefficient of Variation (CV), and agreement in categorical collagen classification was evaluated using Cohen’s kappa (κ). Inter-block reliability of quantitative collagen measurements was assessed using the intraclass correlation coefficient (ICC), calculated using a two-way random-effects model for absolute agreement of single measures (ICC(2,1)), accompanied by its 95% confidence interval (95% CI). Intra-WSI reproducibility was evaluated using ICC(A,1), corresponding to a two-way mixed-effects model for absolute agreement of single measures. Agreement between paired and repeated measurements was further evaluated using Bland-Altman analysis, including calculation of the mean bias and 95% limits of agreement (LoA: mean difference ± 1.96 SD). The relationship between collagen measurements in paired and repeated blocks was assessed using Pearson’s correlation coefficient and visualized with a scatter plot.

A p-value < 0.05 was considered statistically significant.

Statistical analyses were performed using R (version 4.4.2; R Foundation for Statistical Computing, Vienna, Austria) [25] within the RStudio integrated development environment (version 2024.12.1 + 563; RStudio PBC, Boston, MA, USA) [26] employing the following packages: FSA [27], car [28], ggplot2 [29], scales [30], ggpubr [31], irr [32], psych [33], pROC [34] and plotROC [35].

## Results

### Study population and demographics

A total of 69 pituitary adenomas/PitNets met the inclusion criteria for the present study. The cohort comprised 41 males (59.4%) and 28 females (40.6%), with ages ranging from 28 to 82 years (59.5 ± 13.0 years) for males and 30 to 87 years (55.1 ± 15.3 years) for females. Among the 69 pituitary adenomas/PitNets analyzed, 30 (43.5%) were classified as soft, 22 (31.9%) as firm, and 17 (24.6%) as fibrous.

Immunohistochemical analysis revealed the following distribution of pituitary adenomas/PitNets subtypes: gonadotroph tumors were the most prevalent, accounting for 39 cases (56.5%), followed by corticotroph tumors with 7 cases (10.1%), lactotroph tumors with 6 cases (8.7%), null cell tumors with 6 cases (8.7%), mixed tumors with 6 cases (8.7%), and somatotroph tumors with 5 cases (7.3%).

### Digital pathology analysis and collagen quantification

**Fig. 2:**
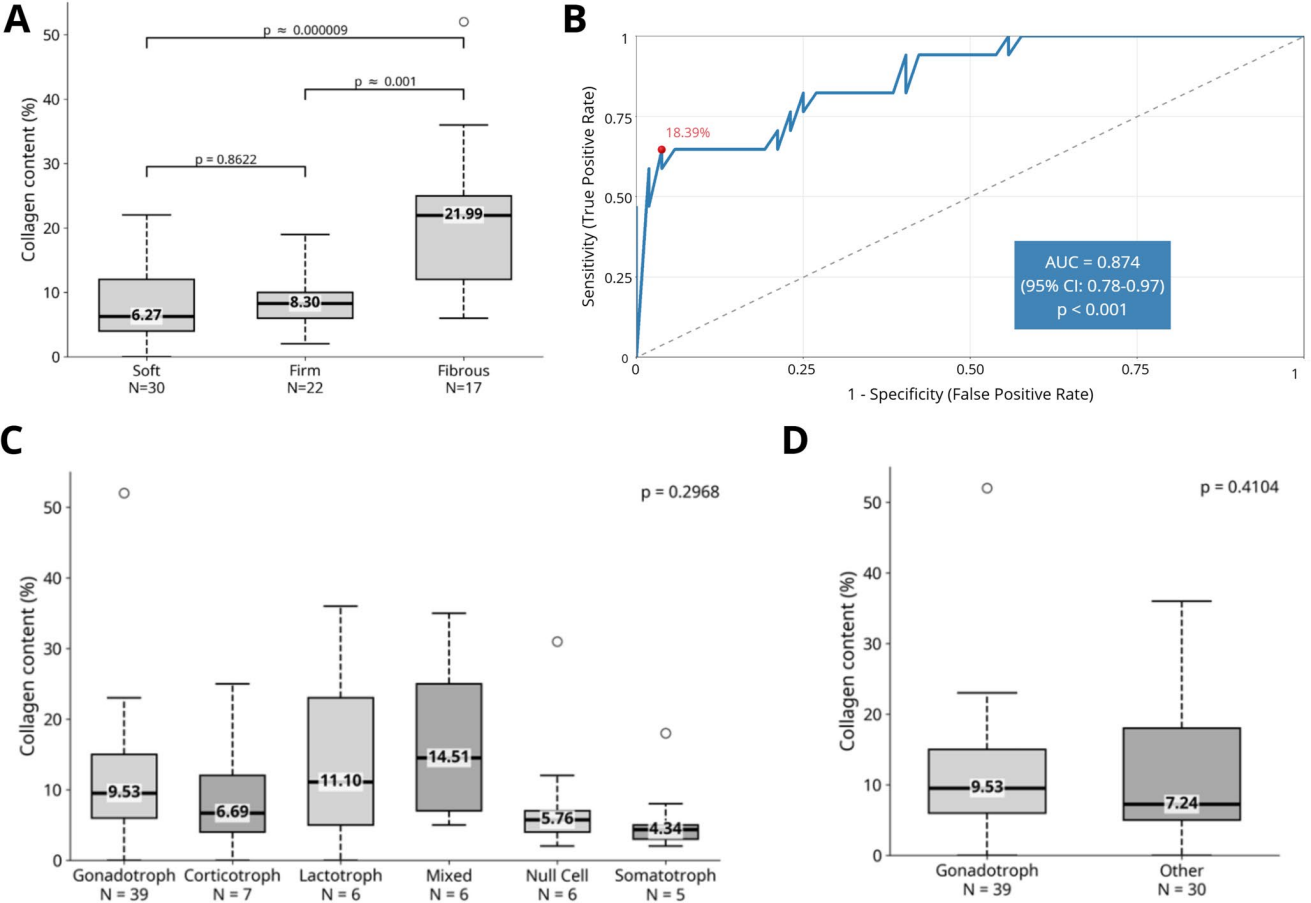
(**A**) Boxplot showing the distribution of collagen percentages, using the QuPath software, across the different consistency groups, with the median value displayed as bold lines. The dot represents an outlier. (**B**) Collagen-based prediction of tumor consistency. Receiver Operating Characteristic (ROC) curve showing the performance of collagen content in predicting tumor consistency (fibrous vs. non-fibrous). Optimal cutoff, 18.39%. (**C**) Boxplots for each pituitary adenomas/PitNets subtype, with medians displayed as bold lines and numerical values provided. (**D**) Boxplots comparing gonadotroph adenomas with all other subtypes. Outliers are represented by dots. P-values are reported in each panel. Sample sizes are shown below each category

Collagen was quantified across all 69 specimens. The median collagen content was 6.27% (IQR: 3.52–9.65%) for soft tumors, 8.30% (IQR: 5.12–12.48%) for firm tumors, and 21.99% (IQR: 15.67–28.31%) for fibrous tumors. Comparative analysis revealed statistically significant differences in collagen content among tumor consistency groups. Collagen levels were significantly higher in fibrous tumors compared with soft (*p* < 0.001) and firm tumors (*p* < 0.001), whereas no significant difference was observed between soft and firm tumors (*p* = 0.862) (Fig. 2A).

### ROC analysis

The ROC curve analysis yielded an AUC of 0.874 (95% CI: 0.779–0.970), indicating a high discriminative power. Based on the Youden Index, the optimal threshold for predicting surgical consistency was established at 18.39%. At this point, our methodology achieved a specificity of 96.15% and a sensitivity of 64.71%. Overall, the model demonstrated a diagnostic accuracy of 86.36% (Fig. 2B).

### Relationship between pituitary adenomas/PitNets subtype and tumor and patient characteristics

Analysis of the association between pituitary adenomas/PitNets subtype and intraoperative consistency revealed no statistically significant relationships. When evaluating each subtype individually, no association with consistency was identified (*p* = 0.540), nor in the comparison between gonadotroph adenomas and all other subtypes combined (*p* = 0.175).

Similarly, no statistically significant differences in collagen content were observed among pituitary adenomas/PitNets subtypes (*p* = 0.297), as illustrated in Fig. 2C. The targeted comparison between gonadotroph tumors and all remaining subtypes likewise demonstrated no significant difference in collagen content (*p* = 0.410) (Fig. 2D).

Linear regression analysis examining the relationship between patient age and collagen content yielded a near-zero Pearson correlation coefficient (*r* = 0.006), indicating no meaningful linear association (*p* = 0.960; r² = 3.7 × 10⁻⁵).

### Inter-block collagen deposition variability experiment and internal validation of the methodology

For the inter-block variability analysis, 22 pituitary adenomas/PitNets were selected from the initial 69 patient cohort. Of these, 10 (45.5%) were classified as soft, 7 (31.8%) as firm, and 5 (22.7%) as fibrous. New tissue blocks were analyzed from the same surgical specimens.

Collagen was quantified across the 22 specimens. Comparative analysis between these paired tissue blocks demonstrated intratumoral heterogeneity in collagen deposition, with a mean CV of 59.83%. No systematic differences were observed between paired samples (*p* = 0.262). Agreement in collagen classification between tissue blocks was moderate (κ = 0.492; *p* < 0.05), although a strong positive correlation was observed between collagen quantification in paired FFPE tissue blocks (*r* = 0.75; *p* < 0.001; 95% CI: 0.47–0.89), indicating a significant linear relationship between measurements.

To perform the retest for internal validation, 6 pituitary adenomas/PitNets were selected randomly from the 69 samples cohort.

In the internal validation of the methodology, the intra-WSI reproducibility analysis showed a high level of agreement between repeated measurements (ICC(A,1) = 0.968; 95% CI: 0.263–0.996) and a near-perfect correlation was also observed (*r* = 0.994; 95% CI: 0.942–0.999; *p* < 0.001).

**Fig. 3:**
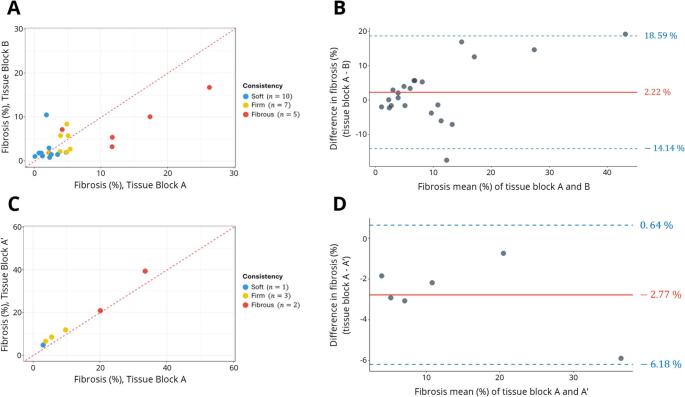
(**A**) Scatter plot showing the relationship between collagen quantification in paired FFPE tissue blocks (A and B) from the same patient. Each dot represents an individual patient, colored according to intraoperative consistency groups (blue, yellow, orange), whose sample size is indicated. The red dotted line represents the line of identity (y = x), indicating perfect agreement between measurements. (**B**) Bland-Altman assessment of inter-block agreement in collagen quantification between paired FFPE tissue blocks (A and B) from the same patient. Each dot represents an individual patient. The red line denotes the mean bias (2.22%), and the blue dashed lines indicate the upper (18.59%) and lower (–14.14%) limits of agreement. (**C**) Scatter plot showing the relationship between collagen quantification in repeated FFPE tissue blocks (A and A’). Each dot represents an individual patient, colored according to intraoperative consistency groups (blue, yellow, orange), whose sample size is indicated. The red dotted line represents the line of identity (y = x), indicating perfect agreement between measurements. (**D**) Bland-Altman assessment of the test-retest agreement in collagen quantification between repeated FFPE tissue blocks (A and A’). Each dot represents an individual patient. The red line denotes the mean bias (–2.77%), and the blue dashed lines indicate the upper (0.64%) and lower (–6.18%) limits of agreement

These findings are further illustrated in Fig. 3, which depicts both agreement (Bland-Altman analysis) and correlation (scatter plot assessment) between paired (Fig. 3A and B) and repeated tissue blocks (Fig. 3C and D).

## Discussion

This study demonstrates a clear and statistically significant relationship between intratumoral collagen content and intraoperative tumor consistency in pituitary adenomas/PitNets. To our knowledge, this is the first study to apply a digital pathology-based approach to objectively quantify collagen content across WSIs, providing a comprehensive and reproducible assessment of the histologic substrate underlying tumor consistency.

Fibrous tumors exhibited markedly higher collagen content than soft or firm lesions, providing objective quantitative support for long-standing clinical observations. No associations were found between collagen content and either pituitary adenomas/PitNets subtype or patient age, suggesting that tumor fibrosis is primarily determined by intrinsic tumor biology. Importantly, this work represents the first comprehensive application of WSI analysis for quantitative assessment of fibrosis in pituitary adenomas/PitNets, effectively overcoming the sampling limitations inherent to previous region-based histological approaches.

Previous histopathological investigations relied on selective high-magnification fields or manually chosen regions, which introduce sampling bias and may fail to capture the heterogeneous distribution of collagen within these tumors. For example, Yiping et al. [36] quantified fibrosis by analyzing five randomly selected fields per case, while Chen et al. [37], Kamimura et al. [38], Wang et al. [15], and Wei et al. [39], used similar region-based approaches under Masson trichrome or Sirius red. Other authors like Li et al. [6] used fluorescence staining (Table 1). Our findings align with the general trend reported in these studies, namely that fibrous tumors contain substantially more collagen. However, the whole-slide methodology implemented here provides more robust and representative fibrosis quantification.

**Table 1:** Comparative methodologies and results from the present and previous studies

Study	*n*	Methodology	Stain	Collagen percentage (%)			*p*-value
				Soft	Firm	Fibrous	
Wang et al. (2009)	38	Five random fields	Picro-Sirius red	8.0 ± 5.2	N/A	21.0 ± 8.4	< 0.01
Wei et al. (2015)	38	Five random fields (×200)	Picro-Sirius red	1.5 ± 0.9	7.4 ± 3.0	18.1 ± 8.2	< 0.01
Yiping et al. (2016)	34	Five random fields (×200)	Masson trichrome	7.3 ± 1.8	N/A	17.7 ± 2.0	< 0.05
Kamimura et al. (2021)	49	Random fields (×400)	Azan trichrome	6.6 ± 3.5	N/A	44.1 ± 15.1	< 0.01
Li et al. (2021)	55	Average optical density values (Image-J)	Immunofluorescence COLI	0.046	N/A	0.052	< 0.05
			Immunofluorescence COLIII	0.044	N/A	0.050	< 0.05
Chen et al. (2024)	290	Five random fields	Masson trichrome	15.6 ± 1.4	N/A	26.1 ± 3.1	< 0.002
Present study	69	Whole slide imaging + AI (QuPath)	Masson trichrome	6.27 (3.52–9.65)	8.30 (5.12–12.48)	21.99 (15.67–28.31)	< 0.05

Values from all previous studies are reported as mean ± standard deviation, while results from the present study are expressed as median and interquartile range

The adoption of WSI analysis with convolutional neural network-based pixel classification represents a significant methodological advance. This approach mitigates the subjectivity inherent to manual microscopy and allows reproducible, operator-independent quantification of collagen across the entire tumor area [40]. Given the often patchy and uneven distribution of fibrosis in pituitary adenomas/PitNets, whole-slide quantification improves accuracy and aligns with the broader transition toward computational pathology in endocrine and neuro-oncological practice. Emerging machine learning approaches in pituitary adenoma/PitNet research, such as the predictive model by Veloso Pereira et al. [41], further highlights the potential of integrating multi-modal data for consistency prediction. In that study, a support vector machine model combining demographic information and quantitative MRI parameters accurately classified pituitary adenomas/PitNets as soft or non-soft, underscoring the clinical utility of interpretable, non-invasive prediction tools for surgical planning.

Tumor consistency remains a critical determinant of surgical strategy and outcomes. In some published series, however, only two categories are typically distinguished (soft and fibrous tumors), with firm and fibrous lesions generally grouped together within the fibrous category [9, 22, 42–45]. Soft tumors are usually amenable to suction-based debulking, whereas fibrous tumors require curettage or sharp dissection, contributing to lower complete resection rates and higher complication risks [9, 46]. The significant differences in collagen content identified here provide quantitative validation for these clinical distinctions, at least between soft and fibrous tumors. The absence of correlation between age and fibrosis reinforces the concept that fibrotic transformation reflects intrinsic tumor characteristics rather than patient-related factors.

Radiological attempts to predict tumor consistency have produced inconsistent results. While Fiore et al. [5] demonstrated an inverse correlation between collagen content and T2-weighted MRI signal intensity, other studies failed to reproduce such associations [21, 36, 42, 47–52]. These discrepancies underscore the challenges of relying solely on imaging and support the development of integrated predictive scores, such as the PiTCon score [7], which combine clinical, radiological, and tumor-related variables.

**Fig. 4:**
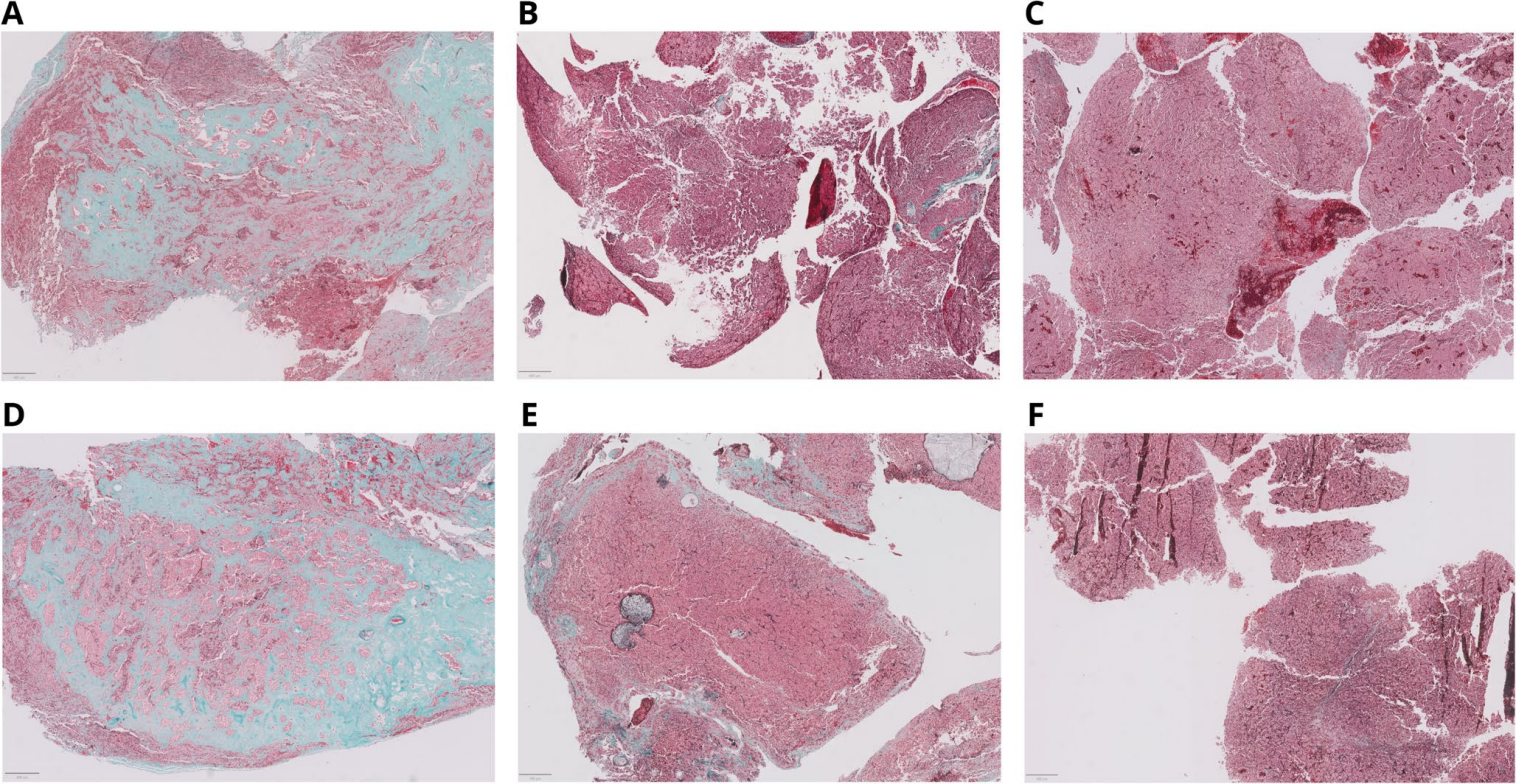
Assessment of intratumoral collagen heterogeneity across paired FFPE tissue blocks. Representative images of three surgical specimens stained with Masson’s trichrome. The first row (**A**-**C**) displays the initial FFPE tissue block, while the second row (**D**-**F**) shows a different block from the same surgical specimen. Scale bar: 400 μm. (A, D) Consistent fibrous pituitary adenoma/PitNet, showing high collagen deposition in both blocks. (B, E) Example of intratumoral heterogeneity. The first block was quantified as soft, whereas the second block reached the threshold for fibrous classification. (C, F) Consistent soft pituitary adenoma/PitNet, with low collagen deposition across both samples

This study presents several methodological strengths that ensure the robustness of our findings. To begin with, intraoperative consistency was assessed by a single senior neurosurgeon throughout the entire cohort. This choice eliminates inter-operator variability as a potential confounding factor. While we acknowledge that this decision may limit generalizability to multi-surgeon settings, the validity of these assessments is independently confirmed by our histological data. A certified neuropathologist, entirely blinded to the surgical consistency data, performed the digital collagen quantification on all 69 specimens. Despite the subjective initial selection of the tumoral area, intratumoral collagen quantification is an objective procedure as it is based solely on the quantification of image pixels within the area of interest. The identification of a strong, statistically significant and monotonically progressive relationship between consistency grades and collagen content (*p* < 0.001) provides an operator-independent confirmation that surgical grading captures genuine biological differences in tumor fibrosis. As well, the high intratumoral heterogeneity observed in our inter-block analysis (CV = 59.83%), highlights the complex and patchy nature of collagen deposition in pituitary adenomas/PitNets. The high variability within FFPE tissue blocks from the same sample justifies the use of WSIs for analyzing entire tissue surface, avoiding the sampling bias inherent in traditional random fields assessment. Our digital pathology approach effectively integrates this biological diversity into a single and representative metric. As a consequence, our model maintains a high diagnostic accuracy (AUC = 0.87; 86.36% of diagnostic agreement) despite such intrinsic heterogeneity, being also able to identify fibrous tumors that may require alternative surgical strategies. Our test-retest analysis demonstrated an excellent degree of methodological reproducibility (ICC(A,1) = 0.968; 95% CI: 0.263–0.996). Despite the fact that the 95% CI is broad due to the limited size of the retested cohort, the high point estimate aligns with the exceptional reliability of the QuPath platform (ICC = 0.992) reported by Acs et al. [53]. All these findings affirm that our methodology delivers stable and consistent measurements, ensuring that the observed variance is attributed to true biological heterogeneity rather than procedural inconsistencies. The visual comparison depicted in Fig. 4 highlights the patchy distribution of fibrosis and the importance of a whole-slide analysis.

Nonetheless, certain limitations should be acknowledged. Although the overall sample size was sufficient to detect differences between consistency groups, subgroup analyses by tumors subtype had limited statistical power. Also, the reliance on a single operator for intraoperative consistency assessment, while reducing variability, may introduce systematic bias. For future perspectives, larger multicenter cohorts should be analyzed to enhance external validity and enable more detailed and robust analysis of pituitary adenomas/PitNets subtypes. A better understanding of the molecular processes within tumor fibrosis would be possible with the inclusion of collagen-type-specific quantification, specifically differentiating collagen types I and III. Furthermore, using radiological markers such as elastography, radiomics, and MRI sequences may aid in the development of multidimensional frameworks for tumor consistency characterization. These multimodal datasets may facilitate the development of predictive models, based on machine learning algorithms, able to enhance preoperative evaluation of patients, and direct individualized surgical approaches in pituitary adenomas/PitNets. In conclusion, this study demonstrates a clear and statistically significant association between intratumoral collagen content and intraoperative tumor consistency in pituitary adenomas/PitNets, providing robust quantitative validation of established surgical classifications distinguishing soft and fibrous tumors. The substantially higher, quantitatively measured collagen levels observed in fibrous tumors reinforce the clinical relevance of distinguishing these tumors during surgical planning, while the absence of associations with pituitary adenomas/PitNets subtype or patient age suggests that the fibrous transformation may be independent of patient-related factors, supporting a tumor-intrinsic origin. Although digital collagen quantification can only be performed postoperatively, except in the uncommon setting of preoperative biopsy, and therefore cannot be used as a standalone method for preoperative prediction, the digital pathology approach applied here offers greater precision than traditional region-based methods and establishes a reliable histological reference standard for future research. These quantitative measurements may serve as ground truth for the development of preoperative predictive models based on radiomics, advanced MRI techniques, and machine learning algorithms designed to identify imaging signatures of tumor consistency. Integrating clinical, radiological, histological and computational features may ultimately yield multimodal predictive tools capable of improving preoperative assessment, guiding surgical strategy, and enhancing individualized management of patients with pituitary adenomas/PitNets.

## Data Availability

The datasets used and/or analyzed during the current study are available from the corresponding author on reasonable request.
